# Mitochondrial DNA 4977 bp Deletion in Peripheral Blood Is Associated With Polycystic Ovary Syndrome

**DOI:** 10.3389/fendo.2021.675581

**Published:** 2021-07-08

**Authors:** Mujin Ye, Bin Hu, Weihui Shi, Fei Guo, Chenming Xu, Shuyuan Li

**Affiliations:** ^1^ International Peace Maternity and Child Health Hospital, School of Medicine, Shanghai Jiao Tong University, Shanghai, China; ^2^ Shanghai Key Laboratory of Embryo Original Diseases, Shanghai, China; ^3^ Shanghai Ji Ai Genetics & IVF Institute, Obstetrics and Gynecology Hospital of Fudan University, Shanghai, China; ^4^ Shanghai WeHealth BioMedical Technology Co., Ltd., Shanghai, China; ^5^ Institute of Reproduction and Development, Obstetrics and Gynecology Hospital, Fudan University, Shanghai, China

**Keywords:** polycystic ovary syndrome, mitochondrial DNA deletion, mitochondrial DNA copy number, mitochondria, infertility

## Abstract

**Background:**

Polycystic ovary syndrome (PCOS) is a common endocrine disorder worldwide. We aimed to examine the associations of two mitochondrial DNA (mtDNA) biomarkers in the peripheral blood, mtDNA copy number (CN), and mtDNA^4977^ deletion rate (DR), with PCOS in a clinical setting.

**Methods:**

We performed a study involving 263 women with PCOS and 326 age-matched controls between June 2015 and June 2019. The mtDNA CN and mtDNA^4977^ DR were measured using multiplex probe-based qPCR. The associations of the mtDNA CN and mtDNA^4977^ DR with the risk of PCOS were estimated using logistic regression.

**Results:**

Analysis of the associations between mtDNA biomarkers and PCOS indicate that the mtDNA CN (*P* = 0.003) and mtDNA^4977^ DR (*P* < 0.001) in PCOS patients were significantly higher than those in the controls. After adjusting for the body mass index, luteinizing hormone/follicle-stimulating hormone ratio, and testosterone level, only higher mtDNA^4977^ DR was associated with PCOS (odds ratio 1.053, 95% confidence interval 1.024 to 1.083; *P* < 0.001). The linear dose-response trends of the mtDNA^4977^ DR were also supported by the quartile analysis.

**Conclusion:**

Multivariable models suggest that mtDNA^4977^ DR levels are strongly associated with PCOS and represent an independent risk factor for PCOS. Further investigation of the utility of mtDNA as a biomarker for PCOS is warranted.

## Introduction

Polycystic ovary syndrome (PCOS), characterized by polycystic ovarian morphology, hyperandrogenism, and/or ovulatory dysfunction, is a common endocrine disorder worldwide. According to the 2003 Rotterdam criteria, the prevalence of PCOS ranges from 4 to 19.9% (1, 2). Women with PCOS are frequently accompanied by infertility, chronic inflammation, and metabolic dysfunction. Consequently, the risk of type 2 diabetes, cardiovascular events, and pregnancy complications (e.g., gestational diabetes, premature birth, and eclampsia) increases in individuals with PCOS (3–5). Over the last few years, increased oxidative stress (OS) has been shown to be involved in the pathophysiology of PCOS (6–8). Previous studies have shown that the levels of circulating markers of OS in women with PCOS were abnormal, independent of weight status (9, 10). In addition, OS has been closely associated with PCOS-related complications, such as insulin resistance, obesity, low-level inflammation, and hyperandrogenism (11–13).

Mitochondria, the powerhouses of cells, regulate cellular ATP production and reactive oxygen species. It has been universally acknowledged that mitochondria are critically involved in the regulation of OS. Mitochondrial DNA (mtDNA) is a circular double-stranded genome, encoding 13 essential proteins involved in oxidative phosphorylation, 2 ribosomal RNAs, and 22 transfer RNAs (14). In contrast to the nuclear genome, mtDNA is present in multiple copies (10–10,000) per cell depending on the cell type. The mtDNA copy number (CN) is a relative measure of mitochondrial content, and its variation has been reported to be associated with some chronic diseases (15). Recently, some studies have shown abnormal mtDNA CN in PCOS patients, suggesting that mtDNA CN variation may be involved in the pathophysiology of PCOS, although the results are conflicting (16–20). In addition to mtDNA CN, deletions in mtDNA are associated with diverse human pathologies (21). The mtDNA 4977 bp (mtDNA^4977^) deletion, which sacrifices approximately one‐third of mtDNA, is the most common deletion in the mitochondrial genome and has been considered as potential biomarker that can reflect the relative integrity and damage of mtDNA (22). Considering the relationship between mtDNA^4977^ deletion and OS (23, 24), we hypothesized that the mtDNA^4977^ deletion rate (DR) is related to PCOS and serves as another potential biomarker for PCOS.

To date, the association between the mtDNA^4977^ DR and PCOS is unknown and the association between the mtDNA CN and PCOS remains controversial. The purpose of this study was to examine the associations between two mtDNA biomarkers in peripheral blood, mtDNA CN and mtDNA^4977^ DR, and PCOS in a large clinical setting.

## Methods

### Participants

This study was conducted at the International Peace Maternal and Child Health Hospital (IPMCH) of Shanghai Jiao Tong University School of Medicine (Shanghai, China). The study population included patients who visited the Reproductive Medicine Centre in the hospital from June 2015 to June 2019. The cases represented approximately 90% of the patients with PCOS who first attended the clinic during the study period. PCOS was diagnosed according to the 2003 Rotterdam criteria, which requires the presence of at least two of the following conditions: (1) ultrasonographic polycystic ovarian morphology, (2) clinical or biochemical hyperandrogenism, (3) Oligo- and/or anovulation (25). Women with oligomenorrhea or hyperandrogenism of other causes, including congenital adrenal hyperplasia, 21-hydroxylase-deficient non-classic adrenal hyperplasia, hyperprolactinemia, Cushing’s syndrome, androgen-secreting tumors, or androgenic/anabolic drug use or abuse, were excluded from this study. During the same period, women who attended the clinic due to male infertility or tubal factor infertility were recruited as controls and matched by age (in 5 y bands) with cases. All the control participants had regular menstrual cycles, normal androgen levels, and no hirsutism, diabetes, galactorrhea, or any endocrine or systemic disease that could affect reproductive physiology. Clinical information, including age and body mass index (BMI), and biochemical parameters, including follicle-stimulating hormone (FSH), luteinizing hormone (LH), estradiol (E_2_), progesterone (P), and testosterone (T) levels, were recorded. Overall, 263 PCOS cases and 326 age-matched controls were enrolled in this study. Peripheral blood was collected when they first came to the Reproductive Medicine Centre, to avoid the effect of drugs or treatment on the biochemical parameters, mtDNA CN, or mtDNA^4977^ DR.

### DNA Extraction

Peripheral blood samples of the participants were collected, processed, aliquoted, and frozen at −80°C according to standardized procedures. Total DNA was extracted using a DNeasy Blood & Tissue Kit (Qiagen, cat. # 69504), following the manufacturer’s instructions. DNA was quantified, and DNA integrity was examined.

### Quantification of mtDNA CN and mtDNA4977 DR

The mtDNA CN and mtDNA^4977^ DR were measured by real-time quantitative polymerase chain reaction (qPCR) using a QuantStudio 7 Flex real-time PCR machine. A multiplex qPCR method, which has been validated by several scientific studies, was used to measure the mtDNA CN and mtDNA^4977^ DR. Briefly, two segments, one in the major arc (mtMajArc) and the other in the minor arc (mtMinArc) of the mitochondrial genome, were used to assess the mtDNA^4977^ DR and mtDNA CN, respectively. TaqMan assays of mtMajArc and mtMinArc are detailed in **Supplementary Table S1**. RNase P (Thermo Fisher, cat. # 4403326) was used as the genomic DNA reference. Real-time PCR was performed under the same cycling conditions as previously described (26). Three technical replicates were performed. The mtDNA CN and mtDNA^4977^ DR were calculated using the following formulae: mtDNA CN = 2^△CT(mtDNA CN)^, where △CT(mtDNA CN) = CT_RNase P_ − CT_mtMinArc_; mtDNA^4977^ DR (%) = 2^△CT(mtDNA4977 DR)^ ×100, where △CT(mtDNA^4977^ DR) = CT_mtMinArc_ − CT_mtMajArc_.

### Statistical Analysis

The study was designed to include all adults with PCOS and age-matched controls who fulfilled the inclusion criteria at a ratio of 1:1.2 during the study period; hence, no formal calculation of the sample size was performed. Data were shown as the median (interquartile range, IQR) for non-normal distributions or the mean ± standard deviation (SD) for normal distributions. The mtDNA biomarkers were transformed using logarithmic conversion. Comparisons of normally distributed variables between groups were performed using unpaired *t* test, whereas comparisons of non-normally distributed ones were conducted using the Mann–Whitney U test. When more than two groups were compared, the Kruskal–Wallis test, followed by Bonferroni’s *post hoc* test, was used. The mtDNA CN and mtDNA^4977^ DR were analyzed as continuous variables and quartiles, with the first quartile as the reference group. Binary logistic regression was performed to assess the associations of the mtDNA CN and mtDNA^4977^ DR with PCOS. Odds ratios (ORs) and 95% confidence intervals (CIs) were reported for each analysis. All statistical analyses were performed using R statistical software, version 3.6.2. A *P*-value of 0.05 or less (two-sided) was considered significantly different.

## Results

### Demographic and Biochemical Characteristics of Cases and Controls

From June 2015 to June 2019, 263 women with PCOS and 326 controls were included in this study. Demographic characteristics and biochemical parameters are summarized in **Table 1**. Significant differences were found between PCOS cases and controls with respect to BMI, FSH, LH, LH/FSH, T, and P (*P* < 0.001 for all comparisons), but not E_2_ (*P* = 0.07).

**Table 1:** Clinical characteristics of women with PCOS and control subjects.

Variable	Control (*n* = 326)	PCOS (*n* = 263)	*P*-value^a^
Age (y)	29.00 (28.00, 33.00)	29.00 (27.50, 31.00)	0.133
BMI (kg/m^2^)	20.80 (19.49, 22.86)	23.80 (21.69, 25.96)	<0.001
FSH (IU/L)	7.70 (6.40, 8.96)	6.80 (5.70, 7.90)	<0.001
LH (IU/L)	4.20 (3.10, 5. 30)	7.90 (4.90, 12.40)	<0.001
LH/FSH	0.53 (0.40, 0.71)	1.16 (0.72, 1.75)	<0.001
E_2_ (pmol/L)	164.00 (119.20, 206.80)	173.00 (125.50, 230.50)	0.07
T (nmol/L)	1.41 ± 0.46	2.24 ± 0.76	<0.001
P (nmol/L)	1.6 (1.1, 2.4)	2.2 (1.7, 2.8)	<0.001

Data are given as median (IQR) or mean ± SD.

IQR, interquartile range; SD, standard deviation.

a p values obtained by comparison of variables between PCOS and control groups using the Mann–Whitney U test.

### Increased Relative mtDNA CN and mtDNA^4977^ DR in PCOS

**Figure 1:**
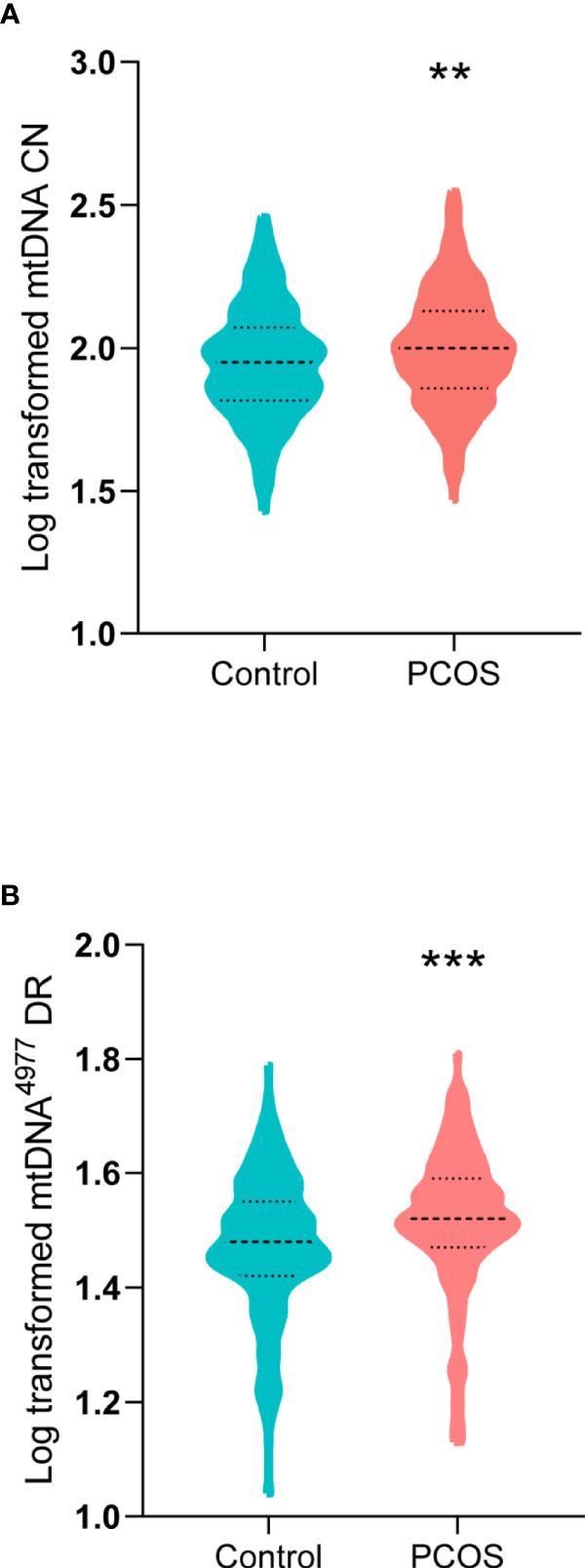
Mitochondrial DNA (mtDNA) copy number (CN) and mtDNA 4977 bp (mtDNA^4977^) deletion rate (DR) in women with PCOS and controls. **(A)** Log-transformed mtDNA CN in women with PCOS and controls (**P = 0.002). **(B)** Log-transformed mtDNA^4977^ DR in women with PCOS and controls (***P < 0.001).

The mtDNA CN and mtDNA^4977^ DR of two groups were compared using the Mann–Whitney U test and Student’s *t* test, respectively. As shown in **Figure 1**, the mean log-transformed mtDNA CN in women with PCOS was significantly higher than that in the controls (2.00 *versus* 1.95, *P* = 0.002). Additionally, the mtDNA^4977^ DR was significantly higher in the PCOS patients than that of controls (1.52 *versus* 1.48, *P* < 0.001).

Further, all subjects were divided into four groups according to the quartiles of the LH/FSH ratio. A significant difference in mtDNA CN as well as mtDNA^4977^ DR was observed among the four groups (**Supplementary Table S2**). After Bonferroni’s correction, a significant difference was confirmed between the lowest and highest quartiles (*P* = 0.011) for the mtDNA^4977^ DR. However, no differences were found among the four groups with respect to the mtDNA CN.

### Associations of mtDNA CN and mtDNA^4977^ DR With PCOS

**Figure 2:**
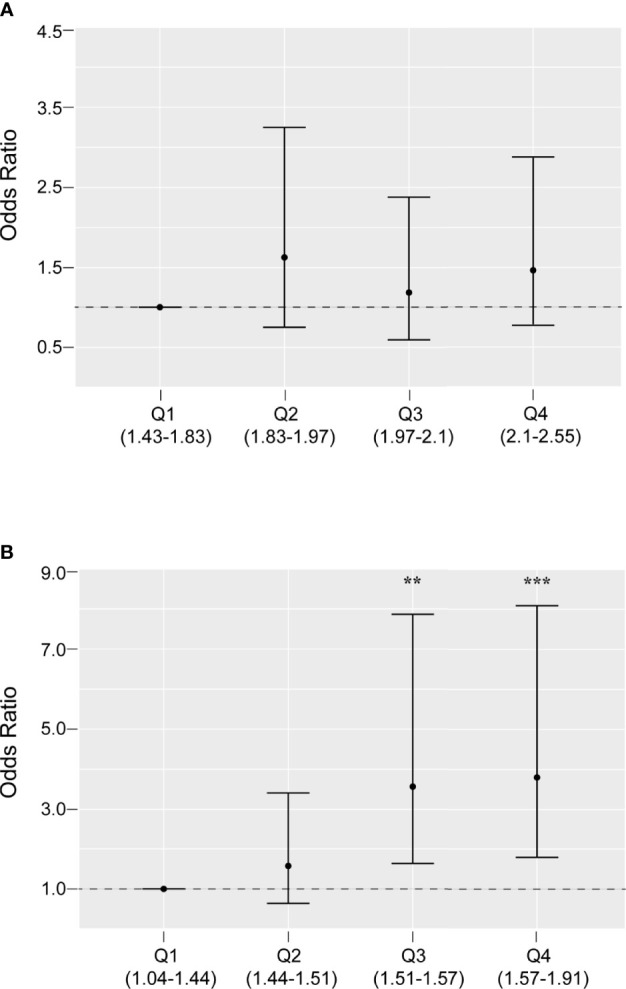
Odds ratios and 95% confidence intervals for PCOS by quartiles of mitochondrial DNA copy number **(A)** and 4977 bp deletion rate **(B)**, adjusted for body mass index, luteinizing hormone/follicle-stimulating hormone ratio, and testosterone level. **P < 0.01, ***P < 0.001

The associations between two potential mtDNA biomarkers and PCOS were assessed using different logistic regression models (**Table 2**). The mtDNA CN was significantly associated with PCOS. However, after adjustment for BMI, LH/FSH ratio, and T level, the mtDNA CN was not associated with PCOS (OR = 1.004, 95% CI: 0.999, 1.008) as a linear continuous variable. This was consistent with the quartile analysis results, which showed no statistically significant associations between the first quartile and higher quartiles (**Figure 2**).

**Table 2:** ORs and 95% CIs for PCOS with mtDNA biomarkers and metabolic parameters.

	OR (95% CI)	*P*-value	Adjusted^†^ OR (95% CI)	*P*-value
mtDNA CN	1.005 (1.002, 1.008)	0.003	1.004 (0.999, 1.008)	0.091
mtDNA^4977^ DR	1.047 (1.027, 1.069)	<0.001	1.053 (1.024, 1.083)	<0.001

mtDNA, mitochondrial DNA; mtDNA^4977^, mitochondrial DNA 4977-bp; CN, copy number; DR, deletion rate; OR, odds ratio; CI, confidence interval.

^†^Adjusted for body mass index, luteinizing hormone/follicle-stimulating hormone ratio, and testosterone level.

Similarly, the mtDNA^4977^ DR was significantly associated with PCOS (*P* < 0.001). In addition, the linear dose-response trends were still apparent in the adjusted model. As shown in **Table 2**, the adjusted OR (95% CI) for PCOS by the mtDNA^4977^ DR was 1.053 (1.024, 1.083). This relationship was also supported by the quartile analysis results (**Figure 2**). Compared with the first quartile, the higher quartiles of the mtDNA^4977^ DR were associated with a higher risk of PCOS, especially the highest quartile (OR = 3.867, 95% CI: 1.847, 8.096). These results imply that the association between the mtDNA^4977^ DR and PCOS is independent of metabolic parameters.

## Discussion

In this study, the elevated mtDNA CN and mtDNA^4977^ DR in the peripheral blood of PCOS patients compared with those in control subjects were observed. However, after adjusting for confounding factors (BMI, LH/FSH ratio, and T level), only the mtDNA^4977^ DR was independently associated with PCOS.

The mtDNA^4977^ deletion, a large deletion of almost one-third of the length of mtDNA, is the most common large deletion in the mitochondrial genome (22). The deleted region contains five tRNA genes and seven genes encoding four complex I subunits, one complex IV subunit, and two complex V subunits. Recently, the mtDNA^4977^ deletion has been shown to be a pathogenic mutation in humans, as it can cause a failure in ATP production and mitochondrial dysfunction (22). Increased OS has been reported to trigger the accumulation of deletions in mtDNA and cause mitochondrial dysfunction (22, 27). Consistent with evidence linking OS with mitochondrial destruction (10, 27), our study indicated that the mtDNA^4977^ DR is a risk factor for PCOS independent of metabolic parameters. Considering the evidence showing that PCOS patients exhibit elevated OS and reduced antioxidant capacity (28, 29), it is plausible to hypothesize that the high level of OS in PCOS may be a potential trigger of mtDNA^4977^ deletion. When the deletion rate reaches a threshold, it causes mitochondrial dysfunction and is involved in the pathogenesis of PCOS. Additionally, it is important to note that the mtDNA^4977^ DR in women with the highest LH/FSH ratio increased significantly. Since an increased LH/FSH ratio has been widely accepted as a specific endocrine profile parameter for many PCOS patients (30), such results could help confirm that a high mtDNA^4977^ DR in peripheral blood is associated with PCOS and that the mtDNA^4977^ DR could serve as a convincing and promising biomarker.

Several studies have suggested that changes in the mtDNA CN are associated with the occurrence and development of PCOS (16, 17, 19). Lee et al. provided the first evidence of decreased peripheral mtDNA CN in PCOS patients (19). Recently, similar results were observed in two other studies, showing that the mtDNA CN in the peripheral blood of PCOS patients was significantly lower than that in healthy controls matched by age and BMI (16, 17). However, the result of our study revealed an elevated mtDNA CN in the peripheral blood of PCOS patients. Consistent with our study, Min et al. detected increased mtDNA CN and increased expression levels of mtDNA replication-related factors in PCOS-patient-derived induced pluripotent stem cells (31). This inconsistency may be attributed to participant preferences or the different sample size of the studied population. In addition, the extent of mtDNA CN alteration was found to be associated with the severity of PCOS (19), indicating that the composition of patients with different severities in different studies might also have influenced the association between the mtDNA CN and PCOS. Considering that this association is not independent of metabolic factors, we speculated that increased mtDNA CN is a compensatory mechanism for mitochondrial dysfunction caused by mtDNA damage, such as mtDNA^4977^ deletion. Further research is warranted to investigate the possible biological mechanisms of abnormal mtDNA CN in PCOS.

To our knowledge, this study is the first to evaluate the association between the mtDNA^4977^ DR and PCOS. The mtDNA^4977^ DR and mtDNA CN were measured simultaneously using an efficient and effective multiplex real-time PCR assay, making our results reliable (26, 32). Additionally, the sample size in this study was larger than that reported in previous similar studies. However, it should be noted that this study was a single-center study based on cross-sectional data in a clinical setting. Multicenter studies are required to validate these findings.

## Conclusion

In summary, the present study demonstrates, for the first time, the contribution of the mtDNA^4977^ DR to PCOS, independent of metabolic parameters. Further study is warranted to elucidate the molecular mechanisms underlying this association.

## Data Availability Statement

The original contributions presented in the study are included in the article/**Supplementary Material**. Further inquiries can be directed to the corresponding authors.

## Ethics Statement

The studies involving human participants were reviewed and approved by Ethics Committee of International Peace Maternal and Child Health Hospital (no. GKLW2018-12). The patients/participants provided their written informed consent to participate in this study.

## Author Contributions

All authors contributed to the article and approved the submitted version. The recruitment of participants and data collection were performed by SL, MY, and BH. The measurement of mtDNA biomarkers and data analysis were performed by BH, WS, and FG. The draft of the manuscript was written by MY and BH. The design of the study and writing review and editing were performed by CX and SL.

## Funding

This work was supported by the National Natural Science Foundation of China (No. 81871136, 81501231, 81971344, and 81771638), the National Key Research and Development Program of China (No. 2016YFC0905103), the International Peace Maternity and Child Health Hospital Clinical Research Project (No. GFY5817 and GFY5818), the Shanghai Municipal Key Clinical Specialty and the Youth Science and Technology Innovation Studio, Shanghai Jiao Tong University School of Medicine.

## Conflict of Interest

Author BH was employed by company Shanghai WeHealth BioMedical Technology Co., Ltd.

The remaining authors declare that the research was conducted in the absence of any commercial or financial relationships that could be construed as a potential conflict of interest.
