# Supplementary material for: Mitochondrial DNA 4977 bp Deletion in Peripheral Blood Is Associated With Polycystic Ovary Syndrome
Source: Front Endocrinol (Lausanne). 2021 Jul 8;12:675581. doi: 10.3389/fendo.2021.675581 (PMC8296803; doi:10.3389/fendo.2021.675581)
Supplement: Supplementary file 1 [file Table_1.docx]

### Supplementary Table

**Supplementary Table S1. Mitochondrial PCR primer/probe sequences.**

|  | **Binding site positions** | **Sequence** |
| --- | --- | --- |
| Major arc |  |  |
| Forward | mt 10 912–10 931 | 5′-CTG TTC CCC AAC CTT TTC CT-3′ |
| Reverse | mt 10 975–10 994 | 5′-CCA TGA TTG TGA GGG GTA GG-3′ |
| Probe | mt 10 934–10 951 | NED- GAC CCC CTA ACA ACC CCC-MGBNFQ |
| Minor arc |  |  |
| Forward | mt 16 528–16 548 | 5′- CTA AAT AGC CCA CAC GTT CCC-3′ |
| Reverse | mt 23–42 | 5′- AGA GCT CCC GTG AGT GGT TA-3′ |
| Probe | mt 16 560–10 | 6FAM-CAT CAC GAT GGA TCA CAG GT-MGBNFQ |

**Supplementary Table S2. The Median mitochondrial DNA (mtDNA) copy number (CN) and mtDNA 4977 bp (mtDNA^4977^)** **deletion rate (DR) by quartiles of luteinizing hormone/follicle-stimulating hormone (LH/FSH) ratio.**

| **LH/FSH**  **(Quartile range)** | **Q1**  **(0.15–0.48)**  **(*n*=147)** | **Q2**  **(0.48–0.68)**  **(*n*=149)** | **Q3**  **(0.68–1.12)**  **(*n*=146)** | **Q4**  **(1.12–4.29)**  **(*n*=147)** | ***p*** ^a^ | | | | | |
| --- | --- | --- | --- | --- | --- | --- | --- | --- | --- | --- |
|  |  |  |  |  | **Q1 vs. Q2** | **Q1 vs. Q3** | **Q1 vs. Q4** | **Q2 vs. Q3** | **Q2 vs. Q4** | **Q3 vs. Q4** |
| **Median mtDNA CN** | 88.94 | 86.91 | 101.68 | 96.57 | 1.000 | 0.162 | 0.587 | 0.075 | 0.313 | 1.000 |
| **Median mtDNA^4977^ DR** | 30.58 | 31.42 | 32.43 | 33.01 | 1.000 | 0.418 | 0.011 | 1.000* | 0.480 | 1.000 |

* Significant differences between groups at the 5% significance level.

^a^ *p* values obtained by comparison of variables between the four quartile groups using the Kruskal-Wallis test, followed by Bonferroni’s correction.
